# The Role of Fc Gamma Receptors in Antibody-Mediated Rejection of Kidney Transplants

**DOI:** 10.3389/ti.2022.10465

**Published:** 2022-07-20

**Authors:** Boris Delpire, Elisabet Van Loon, Maarten Naesens

**Affiliations:** ^1^ University Hospitals Leuven, Leuven, Belgium; ^2^ Nephrology and Renal Transplantation Research Group, Department of Microbiology, Immunology and Transplantation, KU Leuven, Leuven, Belgium; ^3^ Department of Nephrology and Kidney Transplantation, University Hospitals Leuven, Leuven, Belgium

**Keywords:** kidney transplant, renal transplantation, antibody-mediated rejection, AMR, FcγR, FcγR polymorphism

## Abstract

For the past decades, complement activation and complement-mediated destruction of allograft cells were considered to play a central role in anti-HLA antibody-mediated rejection (AMR) of kidney transplants. However, also complement-independent mechanisms are relevant in the downstream immune activation induced by donor-specific antibodies, such as Fc-gamma receptor (FcγR)-mediated direct cellular activation. This article reviews the literature regarding FcγR involvement in AMR, and the potential contribution of FcγR gene polymorphisms to the risk for antibody mediated rejection of kidney transplants. There is large heterogeneity between the studies, both in the definition of the clinical phenotypes and in the technical aspects. The study populations were generally quite small, except for two larger study cohorts, which obviates drawing firm conclusions regarding the associations between AMR and specific FcγR polymorphisms. Although FcγR are central in the pathophysiology of AMR, it remains difficult to identify genetic risk factors for AMR in the recipient’s genome, independent of clinical risk factors, independent of the donor-recipient genetic mismatch, and in the presence of powerful immunosuppressive agents. There is a need for larger, multi-center studies with standardised methods and endpoints to identify potentially relevant FcγR gene polymorphisms that represent an increased risk for AMR after kidney transplantation.

## Introduction

Kidney transplantation remains the most cost-effective treatment for patients with end-stage kidney failure (1). Antibody-mediated rejection (AMR) has been identified as a main reason for this failure (2–5). The term “AMR” defines allograft rejections caused by donor-specific antibodies (DSAs), either against anti-human leukocyte antigens (HLA), blood group antigens, or endothelial cell antigens (6). AMR has been reported to occur in 3%–12% of kidney transplant patients (7) but can be as high as 50% in patients with HLA incompatible transplants (8–10).

Complement-mediated destruction of allograft cells induced by donor-specific anti-HLA antibodies (DSAs) is considered a key component to this pathophysiology of AMR, next to other mechanisms including alternative pathways of NK cell activation and antibody-dependent cellular cytotoxicity (11, 12). C1q binds to the antigen-antibody complexes on the graft endothelium. This activates the complement cascade which ultimately produces a membrane attack complex, initiating osmotic cell lysis. One of the complement split proteins (C4d) can covalently bind to the endothelium or basement membrane collagen. The presence of C4d in the allograft biopsy is therefore regarded as a marker of recent complement activation (13).

However, it was illustrated that graft survival is also impaired in patients with DSAs that are not complement-binding, when compared to patients without antibodies (14, 15). Furthermore, complement-inhibiting therapies did not effectively prevent AMR in all patients with non-complement binding DSAs (16–18). Finally, AMR cases often have no microvascular C4d deposition (19). Taken together, these findings suggest a role of complement-independent processes in antibody-mediated damage of kidney allografts.

Antibodies can also lyse target cells by complement-independent pathways, through the IgG Fc portion and FcγRs variably expressed at the surface of natural killer (NK) cells and of monocytes in a process known as antibody-dependent cell-mediated cytotoxicity (ADCC) (20–25). The antibody Fc region can bind to receptors on monocytes, macrophages, neutrophils, and NK cells. Through interaction between the Fc portion of the coating antibody and the Fc gamma receptor on NK cells, a signalling cascade is initiated that results in the release of cytotoxic granules (containing perforin, granzyme B) and production of cytokines (TNF-alpha and IFN-gamma), ultimately inducing apoptosis of the antibody-coated cell (22).

**FIGURE 1:**
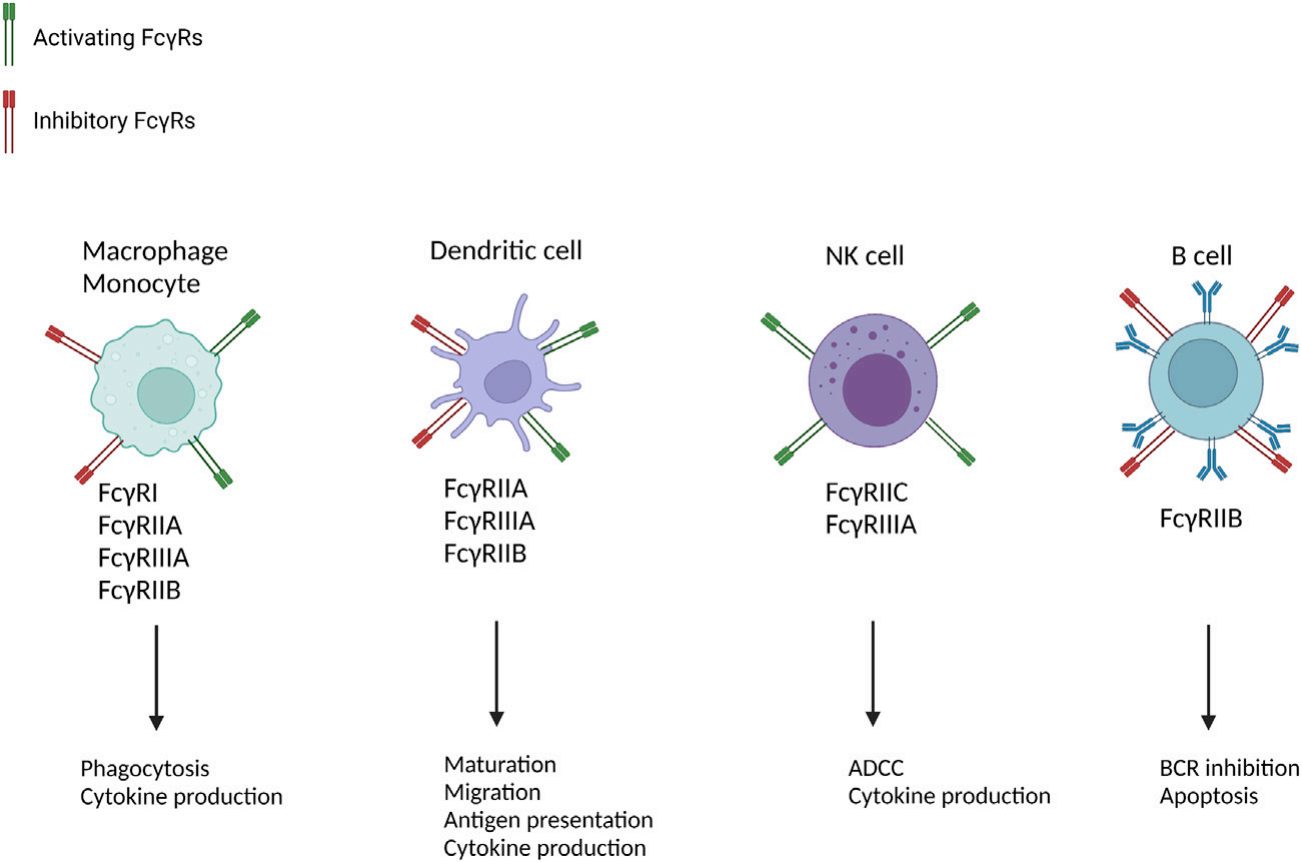
Cellular distribution and function of FcγRs [Adapted from Castro-Dopico et al. (41)]. Multiple immune cells are implicated in AMR and express FcγRs. By promoting cell-type-specific immunological mechanisms they contribute to allograft rejection. B-cells only contain the inhibitory FcγRIIB, which is why they lead to BCR inhibition and apoptosis. NK-cells only express activating FcγRs which is why they only lead to activation of immunological mechanisms such as ADCC and cytokine production. Dendritic cells, macrophages and monocytes contain both activating and inhibitory FcγRs. ADCC, antibody-dependent cellular cytotoxicity; ROS, reactive oxygen species; NET, neutrophil extracellular traps; BCR, B-cell receptor.

There are both inhibiting and activating FcγRs which differ in IgG affinity and signalling mechanisms. These signalling mechanisms can initiate various effector mechanisms including production of reactive oxygen species, cytokines and cytotoxins, immune cell recruitment and activation (Figure 1). Further evidence through histological appearances of FcγR expressing cells in AMR, transcriptomic signatures of FcγRIIIA transcripts in AMR and genetic association studies in transplantation that show a number of single nucleotide polymorphisms (SNPs) in FcγRs, have led to increasing evidence of the major role that FcγRs play in AMR (26–39).

Most SNPs or genetic polymorphisms have no effect on health or disease development, but some of them can act as biological markers by leading to variations in the amino acid sequence of a gene. This way, certain SNPs can be associated with certain diseases or a predisposition to develop a disease later. Several FcγR gene polymorphisms have been shown to change the functionality of FcγRs (29, 39, 40). Decreased immune cell activation, altered binding characteristics to immunoglobulins and altered receptor functions are some examples of how FcγRs can be influenced by certain SNPs. This article reviews the literature on the role of a complement-independent process via FcγRs in the pathophysiology of AMR, and the possible role of FcγR gene polymorphisms in the risk of rejection, AMR and ADCC. In 2016, Castro-Dopico et al reported on this topic (41). We re-evaluated the literature, including more recent references.

## Materials and Methods

A comprehensive literature search was performed by utilizing the following databases: PubMed, Embase and Web of Science core collection.

Our PubMed/MEDLINE search string consisted of the following terms: (“Receptors, IgG”[Mesh] OR “FcγR IIA” [Supplementary Concept] OR “FcγR IIB” [Supplementary Concept] OR “FcγR IIC” [Supplementary Concept] OR “FCΓR3A protein, human” [Supplementary Concept] OR “FCΓR3B protein, human” [Supplementary Concept] OR “FCΓR1A protein, human” [Supplementary Concept] OR “FcγR1 protein, mouse” [Supplementary Concept] OR “FcγR3 protein, mouse” [Supplementary Concept] OR “FcγR2a protein, rat” [Supplementary Concept] OR “Fcγ” OR “Fc gamma” OR “Fcgamma”) AND (“Graft Rejection”[Mesh] OR [(“transplant*” OR “graft*”) AND “reject*”)] AND (“kidney” OR “renal”). 55 hits were found on 14/04/2021.

Our Embase search string consisted of the following terms: “Fc receptor”/exp OR “Fc receptor”: ti, ab, kw OR “Fc receptor IIa”/exp OR “Fc receptor Iib”/exp OR “Fc receptor Iic”: ti, ab, kw OR “fc fragment receptor”: ti, ab, kw OR “FcγR”: ti, ab, kw OR “IgG fc receptor”: ti, ab, kw OR “immunoglobulin fc fragment receptor”: ti, ab, kw OR “immunoglobulin g fc receptor”: ti, ab, kw OR “lymphocyte fc receptor”: ti, ab, kw OR “FcγR”: ti, ab, kw OR “FCΓR1A protein, human”: ti, ab, kw OR “Fc gamma”: ti, ab, kw OR “FCΓR3B protein, human”: ti, ab, kw OR “Fcγ”: ti, ab, kw AND “graft rejection”/exp OR “allograft rejection”: ti, ab, kw OR “graft reaction”: ti, ab, kw OR “allograft reaction”: ti, ab, kw OR “transplant* rejection”: ti, ab, kw AND “kidney”/exp OR “Renal”: ti, ab, kw. 70 hits were found on 07 March 2021.

Our Web of Science core collection search string consisted of the following terms: TS=(“Fc receptor” OR “Fc receptor IIa”/exp OR “Fc receptor IIb”/exp OR “Fc receptor IIc” OR “fc fragment receptor” OR “FcγR” OR “IgG fc receptor” OR “immunoglobulin fc fragment receptor” OR “immunoglobulin g fc receptor” OR “lymphocyte fc receptor” OR “FcγR” OR “FCΓR1A protein, human” OR “Fc gamma” OR “FCΓR3B protein, human” OR “Fcγ”). TS = (“graft rejection” OR “allograft rejection” OR “graft reaction” OR “allograft reaction” OR “transplant* rejection”). TS = (“kidney” OR “Renal”). 47 hits were found on 07 March 2021.

### Study Selection

Articles from databases were identified and selected applying subsequent steps:

1) Identification of titles of records through database searching
2) Removal of duplicates
3) Screening and selection of abstracts. Abstracts had to contain information regarding both FcγRs and kidney transplant rejection (preferably AMR).
4) Judgement for eligibility through full-text articles; texts had to contain a thorough description of an FcγR polymorphism and AMR. They needed to report the incidence of the polymorphism comparing kidney transplant recipients with rejection to kidney transplant recipients without rejection.
5) Final inclusion in study.

After careful consideration, only five articles were included in the review. Multiple reviews and other articles were used to provide a framework and to refer to.

## Results

### Fc-Gamma Receptor and Their Mechanisms of Action

FcγRs are glycoproteins that can be found on the surface of hematopoietic cells and bind to the Fc portion of IgG antibodies. This facilitates a link between the humoral and cellular immune systems (42). The family of FcγRs is involved in antigen presentation, regulation of B cell activation and initiation of intracellular signalling pathways which subsequently lead to immune cell activation and maturation (43). Classical FcγRs include an inhibitory receptor (FcγRIIB) and multiple activating receptors (FcγRI, FcγRIIA, FcγRIIC, FcγRIIIA, and FcγRIIIB).

FcγRs have binding affinity for IgG and can recognize IgG-coated targets, such as opsonized pathogens or immune complexes. After cross-linking of activating FcγRs, tyrosine on the immunoreceptor tyrosine-based activation motif (ITAM) gets phosphorylated. Due note that cross-linking of FcγRs only occurs with aggregated IgG, such as opsonised cells or immune complexes, rather than monomeric IgG (44). Then both Src-kinases Lyn and subsequent recruitment of SH2-containing kinases are responsible for activating ITAM by phosphorylation. ITAMs are located either on the intracellular domain of the FcγRs (e.g., FcγRIIA) or in the associated common γ-chain (e.g., FcγRIIIA). ITAM-P leads to key recruitment of SH2 domain containing kinases, most notably spleen tyrosine kinase (SYK), and the subsequent activation of multiple downstream signalling mediators, including PI3K and PLCγ. All this leads to triggering protein kinase C (PKC) and initiating calcium flux (44, 45). The subsequent mechanisms differ between the different types of immune cells that express FcγR (Figure 1). Differences in these domains account for differences in function of FcyR. In contrast to activating FcγRs, FcγRIIB (inhibitory receptor) contains an intra-cellular immunoreceptor tyrosine-based inhibitory motif (ITIM). Cross-linking of FcγRIIB with activating FcγR leads to Src kinases phosphorylating ITIM and recruiting of inositol phosphatases to neutralise the activating signals (46). Therefore, the FcγRIIB can act as a supplementary regulatory mechanism and suppresses IgG-mediated inflammation (27).

Four different IgG subclasses in humans (IgG1-IgG4) are responsible for the action mechanism of FcγRs. The four IgG subclasses express different affinities to different receptors. IgG1 and IgG3 can efficiently activate the classical route of complement, while IgG2 and IgG4 do this less efficiently or only under certain conditions, as seen with IgG2. This can be explained by the reduced binding of C1q to IgG2 and IgG4 (47).

FcγRs are broadly expressed by hematopoietic cells such as natural killer (NK) cells, mast cells, macrophages, dendritic cells, neutrophils, monocytes, endothelial cells and B-cells (44). Cells can vary in the expression of different types of FcγRs and the levels of expression of these FcγRs, allowing them to modulate the activation threshold when interacting with immune complexes (48). The activation state of FcγR-expressing cells is tightly controlled by the balance between activating and inhibitory FcγR, with the exception of NK cells (49). NK cells express only FcγRIIIA and no inhibitory FcγR. The distribution of the FcγRs across different cell types is illustrated in Figure 1. FcγR-ligated immune cells can directly activate the endothelium by binding to DSA and cause AMR through ADCC without interference of the complement-pathway.

#### Monocytes/Macrophages

Monocytes are innate immune cells that work as potent phagocytes and that can further differentiate into either macrophages or dendritic cells (50). Several studies suggest that monocyte infiltration is a key component of AMR after transplantation (34, 51, 52).

Macrophages express FcγRIIA, FcγRIIIA and FcγRIIB, with the activating FcγRs being more dominantly expressed. Activation of FcγRs leads to phagocytosis and cytokine release (TNF, IL6, IL-1alpha and neutrophil chemoattractants). These responses are counteracted by the inhibiting FcγRIIB (53). In dendritic cells this inhibiting FcγRIIB is dominantly expressed and suppresses immune-complex-mediated pro-inflammatory cytokine release, T-cell stimulation and migration (54–56).

#### Neutrophils

Human neutrophils express both FcγRI, FcγRIIA and FcγRIIIB. Activation of FcγRs on neutrophils leads to increased neutrophil adhesion to endothelial cells, cytokine and superoxide production, phagocytosis and neutrophil extracellular trap formation (NETosis) (57–61). When neutrophil infiltration in AMR is present, they are typically found in peritubular capillary lumens (62, 63).

#### Natural Killer Cells

NK cells primarily express activating FcγRIIIA and in some individuals a small fraction of NK cells may express FcγRIIC (64). As they do not express inhibitory FcγR, they could be the dominant effector cell in ADCC (65). When stimulated through their FcγR, they produce monocyte chemo-attractants CCL3, CCL4 and three effector cytokines; IFN-y, TNF and CSF2 (66).

#### B-Cells

The inhibitory FcγRIIB is the only FcγR expressed by B-cells. After crosslinking with B-cell receptors, the B-cell activation threshold will increase and suppress further antibody production (27).

#### Other Cell Types

Eosinophils express FcγRI, FcγRIIA, FcγRIIB and FcγRIIIB. Binding to antibodies induces degranulation. Platelets express FcγRIIA. Mast cells express FcγRIIB and FcγRIIIB. The role of eosinophils, platelets and mast cells seems limited in the process of AMR.

### Different Fc-Gamma Receptor Polymorphisms Associated With Antibody-Mediated Rejection

Genetic variation in the genes of human FcγRs can alter receptor expression, function and affinity to IgG (27, 67). FcγR single nucleotide polymorphisms (SNPs) are now considered a hereditary risk factor for infectious and autoimmune diseases (68, 69). Also in allo-immune processes, genetic variations in FcγR genes could lead to different susceptibility to AMR. FcγRI has three non-synonymous SNP mutations (rs7531523, rs12078005, and rs142350980) but no studies investigating the association of these polymorphisms with AMR have been published (70). Furthermore, FcγRIIC has one SNP in intron 7 which has an effect on clearance of parasitaemia, but no studies have been published regarding the link with AMR (71). As there is currently no literature available on their association with AMR, they are not further discussed in this literature review.

#### FcγRIIA

FcγRIIA is a key FcγR for IgG-mediated responses in macrophages, monocytes or monocyte-derived dendritic cells (3, 72). FcγRIIA can also be found on the surface of neutrophils, platelets, basophils, eosinophils and other cells (73). The FcγRII gene is located on chromosome 1q23. Genetic variation in this gene locus is linked with several autoimmune and inflammatory diseases (68). The best-studied functionally relevant SNP, rs1801274, has been described in the extracellular domain of FcγRIIA, and exchanges adenine (A) to guanine (G) in the coding region in exon 4 of chromosome 1 (q23-24). As a result, histidine (H) is switched into an arginine (R) amino acid at position 131 in the immunoglobulin-like domain (H/R131), leading to altered receptor affinity and specificity (29). In contrast to FcγRIIIA, FcγRIIA polymorphisms seems to have less effect on AMR outcomes. This difference could be explained by the higher affinity of FcγRIIA for IgG1 instead of IgG3, opposite to the affinity observed in FcγRIIIA polymorphisms (74). The lack of inhibitory receptors on NK cells, who primarily express FcγRIIIA and lack inhibitory FcγRIIB expression, could contribute further to this observation (75).

Three studies investigated the association between the allelic frequency of this FcγRIIA H/R131 polymorphism in recipients with stable graft function compared to kidney transplant recipients with rejection (Table 1). First, Pawlik et al. conducted a case-control study in a population of 82 renal transplant recipients and found that the R/R131 genotype was associated with longer graft survival, which they hypothesized to be mechanistically explained by a lower affinity of this FcyR and less cytokine release, leading to a decreased immune response (39). The probability of graft survival over 7 years was 1.74-fold greater among subjects with the R/R131 polymorphism, compared to the H/H131 polymorphism. Next, and in contrast with their previous results, Pawlik et al. conducted another case-control study of 121 renal transplant recipients and found no significant differences in allele frequency between recipients with chronic rejection and recipients with stable graft function (28). However, Yuan et al., showed a significant positive association of the R/R131 genotype with acute kidney rejection (29). When homozygous, higher trends towards acute rejection were also observed. They noted that only 9 out of 46 (20%) non-rejectors had the FcγRIIA homozygote R/R131 polymorphism, compared to 24 out of 53 (45%) rejectors having the R/R131 polymorphism. The frequency of the R/R131 polymorphism was thus significantly higher in the rejector group compared to the non-rejector group. Finally, a recent large multicentre retrospective study with 1,940 kidney transplant recipients, found no association between the FcγRIIA H/R131 polymorphism and death-censored graft survival, graft function or requirement of rejection treatment (76). This study comprised an unselected cohort analysis with a patient cohort derived from the Collaborative Transplant Study (CTS, www.ctstransplant.org).

**TABLE 1:** Distribution of the FcγRIIA genotypes and allele frequencies in patients with vs. without rejection. Numbers are noted as follows: X/Y (%). X = the number of patients with the specific polymorphism; Y = the total number of patients (study recipients or control population); % = the fraction is calculated to the percentage of people who carry the polymorphism; NS = not significant, X = the number of patients with the specific polymorphism. The *p*-value reflects the significance in differences of the allele frequencies between cases and controls.

	H/H131		H/R131		R/R131		*p*-value	Type of rejection
	Cases	Controls	Cases	Controls	Cases	Controls		
Yuan et al. (29) (Case-control study)	7/53 (13%) kidney transplant recipients with acute rejection	13/46 (28%) recipient non-rejectors	22/53 (42%)	24/46 (52%)	24/53 (45%)	9/46 (20%)	*p* < 0.005	Acute kidney rejection
								No DSA information present
Pawlik et al. (28) (Case-control study)	19/68 (27.9%) kidney transplant recipients with chronic allograft rejection	16/53 (30.2%) recipient non-rejectors	35/68 (51.5%)	26/53 (49.1%)	14/68 (20.6%)	11/53 (20.7%)	NS	Chronic kidney graft rejection
								No DSA information present
Wahrmann et al. (76) (Unselected cohort study)	55/229 (24%) kidney transplant recipients showing need of rejection treatment during 1 year in a cohort of 1010 kidney transplant recipients	206/781 (26.4%) kidney transplant recipients showing no need of rejection treatment during 1 year in a cohort of 1010 kidney transplant recipients	127/229 (55.5%)	412/781 (52.8%)	47/229 (20.5%)	163/781 (20.9%)	*p* = 0.69	Recipients treated for rejection within the first year after transplantation
								No DSA information present

#### FcγRIIIA

FcγIIIA (CD16) is expressed on monocytes/macrophages, dendritic cells, and NK-cells. FcγRIIIA is the only human activating FcγR that has a preferential binding to IgG3. In kidney transplantation, it is suggested that IgG3- DSA positive recipients show more intense microvascular inflammation (77). These findings further suggest the key role of NK cells, monocytes and macrophages in orchestrating the inflammation observed in AMR and may also be, at least in part, the culprits behind the more damaging effects seen with complement-fixing HLA antibodies (15). This further contributes to our hypothesis that different effector mechanisms together lead to graft loss, and not complement-activation alone.

A functional SNP (rs396991) in the gene of FcγRIIIA substitutes a valine (V) to phenylalanine (P) amino acid at position 158 (V/F158), alters the affinity to IgG1 and IgG3 and thus influences immune cell activation (74, 78). For example, Arnold et al. described greater frequency of peritubular capillaritis when the FcγIIIA V158 allele was present due to greater immune cell recruitment in peritubular capillaries (79). Two studies discussed the association between the FcγRIIIA V/F158 polymorphism and AMR after kidney transplantation (Table 2). A case-control study by Litjens et al. linked the V-allele to an increased expression of FcγRIIIA on NK cells and to an increased glomerulitis score in a study of 141 kidney transplant patients (40). Confirming the earlier associations seen in Arnold et al. (79), they observed an association between V-allele and decreased kidney allograft survival after diagnosis of chronic AMR, but the 158V/V genotype itself did not appear to be a risk factor for the development of chronic AMR. Other than the positive association of this polymorphism and increased risk of graft failure after diagnosis of chronic AMR (40), also in heart and lung transplantation clinical associations of cardiac allograft vasculopathy and acute lung transplant rejection with FcγRIIIA polymorphisms have been observed (80, 81). This association between the V/F158 SNP in FcγRIIIA and increased risk of graft failure could be mediated by target cells opsonizing IgG antibodies to bind to FcγRIIIA on immune cells, followed by the release of cytotoxic granules which trigger apoptosis of the target cells. FCGR3A gene expression is also increased in biopsies diagnosed with AMR (36–38). Especially NK cells, which do not express the inhibitory FcγRIIB and thus cannot compensate for overactive FcγRIIIA signalling, could be major contributors to the deleterious effect of this polymorphism.

**TABLE 2:** Distribution of the FcγRIIIA genotypes and allele frequencies in patients with vs. without rejection. Numbers are noted as follows: X/Y (%). X = the number of patients with the specific polymorphism; Y = the total number of patients (study recipients or control population); % = the fraction is calculated to the percentage of people who carry the polymorphism; NS = not significant, X = the number of patients with the specific polymorphism. The *p*-value reflects the significance in differences of the allele frequencies between cases and controls.

	V/V158		V/F158		F/F158		*p*-value	Type of rejection
	Cases	Controls	Cases	Controls	Cases	Controls		
Litjens et al. (40) (Case-control study)	21/133 (15.8%) kidney transplant recipients with c-aAMR	17/116 (14.7%) recipient non-rejectors	59/133 (44.4%)	46/116 (48.7%)	53/133 (39.8%)	53/116 (45.7%)	*p* = 0.65	Chronic active AMR.
								DSA information present
Wahrmann et al. (76) (Unselected cohort study)	29/229 (12.7%) kidney transplant recipients showing need of rejection treatment during 1 year in a cohort of 1010 kidney transplant recipients	105/781 (13.4%) kidney transplant recipients showing no need of rejection treatment during 1 year in a cohort of 1010 kidney transplant recipients	104/229 (45.4%)	350/781 (44.8%)	96/229 (41.9%)	326/781 (37.4%)	*p* = 0.85	Recipients treated for rejection within the first year after transplantation
								No DSA information present

Despite these first suggestions of a significant association between the FcγRIIIA V/F158 polymorphism and AMR and outcome after kidney transplantation, a more recent and larger study included 1940 kidney transplant recipients (76). This study could however not confirm any association of the FcγRIIIA V/F158 polymorphism and impaired allograft function or increased need for rejection treatment within the first year after transplantation. Also in a subanalysis in 438 patients with higher risk of AMR, there was no association of Fc*γ*RIIIA polymorphisms with 10-year death-censored graft survival in this subgroup. We do note that Wahrmann et al. didn’t specifically investigate different mechanisms responsible for allograft loss, like microvascular inflammation, whereas Litjens et al. did (40, 76).

#### FcγRIIIB

FcγRIIIB is expressed on neutrophils and eosinophils. The main function of FcγRIIIB is immune cell clearance of all cells that contain immunoglobulins recognized by FcγRIIIB. By triggering internalisation of captured immune complexes, degradation of antigen-antibody complexes can occur (44). Four amino acid substitutions lead to differences in glycosylation resulting in a FcγRIIIB NA1/NA2 polymorphism. NA1 is more efficient in binding to immune complexes containing IgG1 and IgG3 than NA2 and reduced binding affinity of NA2 genotype could potentially mean that clearance of immune complexes may be reduced (82–85). Furthermore, NA2/NA2 homozygotes show a lower capacity to mediate phagocytosis (86, 87). Because the expression of FcγRIIIB is limited to neutrophils and eosinophils, an association with FcγRIIIB polymorphisms and AMR is not expected. This is due to the fact that neutrophils are rarely observed in late AMR (79). Two studies investigated the difference in incidence of this polymorphism in FcγRIIIB between kidney transplant recipients with stable graft function and kidney transplant recipients with rejection (76, 88) (Table 3). First, a case-control study by Xu et al. showed that NA1/NA2 genotype frequency and allele frequency were not related to acute rejection vs. well-functional grafts in kidney transplant recipients. More recently, Wahrmann et al. confirmed the lack of association between the FcγRIIIB NA1/NA2 polymorphism and death-censored kidney graft survival, graft function or requirement of rejection treatment, in a large cohort of 1,940 kidney transplant recipients.

**TABLE 3:** Distribution of the FcγRIIIB genotypes and allele frequencies in patients with vs. without rejection. Numbers are noted as follows: X/Y (%). X = the number of patients with the specific polymorphism; Y = the total number of patients (study recipients or control population); % = the fraction is calculated to the percentage of people who carry the polymorphism; NS = not significant, X = the number of patients with the specific polymorphism. The *p*-value reflects the significance in differences of the allele frequencies between cases and controls.

	FcγRIIIB (NA1/NA1)		FcγRIIIB (NA1/NA2)		FcγRIIIB (NA2/NA2)		*p*-value	Type of rejection
	Cases	Controls	Cases	Controls	Cases	Controls		
Xu et al. (88) (Case-control study)	9/85 (10.6%) kidney transplant recipients with AMR or cellular rejection	11/86 (11%) recipient non-rejectors	60/85 (70.6%)	61/86 (70.9%)	16/85 (18.8%)	14/86 (16.3%)	*p*=NS	Acute AMR or cellular kidney rejection
								No DSA information present
Wahrmann et al. (76) (Unselected cohort study)	30/229 (13.1%) kidney transplant recipients showing need of rejection treatment during 1 year in a cohort of 1010 kidney transplant recipients	87/781 (11.1%) kidney transplant recipients showing no need of rejection treatment during 1 year in a cohort of 1010 kidney transplant recipients	108/229 (47.2%)	349/781 (44.7%)	91/229 (39.7%)	345/781 (44.2%)	*p* = 0.20	Recipients treated for rejection within the first year after transplantation
								No DSA information present

#### FcγRIIB

FcγRIIB is the only inhibitory FcγR and can be found on B cells, mast cells, macrophages, neutrophils, and eosinophils. The rs1050501 SNP induces a threonine to isoleucine substitution at position 232. Because this occurs within the transmembrane domain of the receptor, FcγRIIB I/T232 is responsible for the dysfunction of the receptor (89, 90). Dysfunction of this inhibitory receptor could theoretically lead to increased immune activation and associations with several autoimmune diseases have been found such as systemic lupus erythematosus, MS and ITP (87, 91–94). Murine studies previously showed associations between FcγRIIB I/T232 and outcomes on kidney allograft by raising the susceptibility to develop chronic AMR (95), but these results could not be replicated in a large human study by Clatworthy et al. (96). They conducted an analysis of three cohorts of 2,851 Caucasian transplant recipients, 570 Afro-Caribbean transplant recipients and 236 patients with a diagnosis of SLE derived from the CTS (96). No association could be found between presence of the FcγRIIB I/T232 polymorphism and differences in 10-year transplant survival. This contradiction could be explained by the observation that expression, structure, associated signalling molecules and most importantly, affinity for different IgG subclasses differ between murine and human FcγRs (97–99). They do however note that their failure to detect an association could be because their effect size of this SNP is smaller than estimated by their power calculations (96). An increased number of patients in a follow-up study could more accurately detect differences or further prove that no associations can be found.

## Discussion

Antibody-dependent cellular cytotoxicity is considered to play a major role in the pathophysiology of AMR after kidney transplantation, through the involvement of FcγRs. The mechanism of action and cellular expression of these receptors is well known. Several functional SNPs have been described in these FcγRs and could theoretically impact the risk of AMR after kidney transplantation. Although several studies have addressed this question, it remains however difficult to make conclusions about the role of FcγRs polymorphisms in the risk of AMR. Earlier and smaller studies (28, 30, 40, 88) described associations between FcγR polymorphisms and microcirculation inflammation. However, Wahrmann et al. did not confirm associations between these Fc*γ*R gene variants and early rejection, graft function, or long-term allograft failure (76). Even in patients who were sensitised and thus at higher risk for AMR, no associations were found with transplant outcomes.

The discrepancy between the studies are primarily explained by the wide heterogeneity in the choice and definition of the primary endpoints (graft dysfunction, acute and chronic rejection, graft survival time, …), which make comparisons between the studies complex. If for instance the rejection subtype is not evaluated, as was the case for Wahrmann et al. (76), it could be that potential associations between polymorphisms and subtypes of rejection are missed. Other sources of heterogeneity include demographic differences between the cohorts, differences in study design, different background immunological risk of the included patients, numbers of centres, era, etc. Study populations were overall rather small with the exception of the studies by Clatworthy et al. and Wahrmann et al. (76, 96). Also, when AMR is studied, detailed information on DSA is necessary, which is often not available (100). This is a major limitation of the literature on this topic, which importantly hampers making strong conclusions on the association of FcγR polymorphisms and AMR. This could explain why most studies, including Wahrmann et al., have failed to find any associations, while studies where detailed DSA information was available described significant associations between FcγR polymorphisms and the risk of prognosis of AMR. More systematic research on larger-scale collaborative cohorts, and detailed phenotyping of the cases are needed.

In conclusion, our literature review indicates a role of FcγRs in kidney transplant rejection, and the theoretical relevance of the FcγRs polymorphisms in AMR after kidney transplantation. However, in the absence of robust and sufficiently detailed and large-scale studies assessing the actual association of the polymorphisms with well-defined clinical events, we cannot make any robust conclusion on the clinical relevance of these polymorphisms. Furthermore, the two largest, multicenter studies, could not provide evidence for functional FcγR polymorphisms and therefore no impact on incidence of AMR. More systematic large and multi-center studies are needed to robustly determine the potential role of FcγR polymorphisms in the risk of AMR after kidney transplantation, independent of clinical risk factors and the donor-recipient genetic mismatch and in presence of potent immunosuppressive agents, but most importantly, with notion of DSA present.

## Abbreviations

AMR, antibody-mediated rejection; APC, antigen-presenting cell; DSA, donor-specific antibodies; FcγR, Fc-gamma receptor; IFNγ, interferon gamma; HLA, human leukocyte antigen; ITAM, immunoreceptor tyrosine-based activation motif; ITIM, immunoreceptor tyrosine-based inhibitory motif; MHC, major histocompatibility complex; NK, natural killer; PI3-K, phosphatidylinositol3-kinase; PLCy, phospholipase Cy; PKC, protein kinase C; SNP, single nucleotide polymorphism; TCMR, T-cell mediated rejection.
